# Reporting Modifications in Surgical Innovation: A Systematic Scoping Review Protocol

**DOI:** 10.29337/ijsp.167

**Published:** 2021-11-12

**Authors:** Christin Hoffmann, Sina Hossaini, Sian Cousins, Natalie Blencowe, Angus G. K. McNair, Jane M. Blazeby, Kerry N. L. Avery, Shelley Potter, Rhiannon Macefield

**Affiliations:** 1 National Institute for Health Research Bristol Biomedical Research Centre (Surgical Innovation Theme), Centre for Surgical Research, Bristol Medical School: Population Health Sciences, University of Bristol, Bristol, UK; 2 University Hospitals Bristol and Weston NHS Foundation Trust, Bristol, UK; 3 North Bristol NHS Trust, Bristol, UK

**Keywords:** Innovation, Surgery, Modifications, Review, Operative, Procedures

## Abstract

**Introduction:**

Innovation in surgery drives improvements to patient care. New surgical procedures and devices typically undergo a series of modifications as they are developed and refined during their introduction into clinical practice. These changes should ideally be reported and shared between surgeon-innovators to promote efficient, safe and transparent innovation. Currently, agreement on how modifications should be defined, conceptualised and classified, so they can be reported and shared efficiently and transparently, is lacking. The aim of this review is to examine and summarise existing literature on definitions, perceptions and classifications of modifications to surgical procedures/devices, including views on how to measure and report them. The findings will inform future work to standardise reporting and sharing of modifications in surgical innovation.

**Materials and Methods:**

A systematic scoping review will be conducted adhering to PRISMA-ScR guidelines. Included articles will focus on review articles and opinion pieces relevant to modifications to new surgical procedures or devices introduced to clinical practice. Methods to identify relevant literature will include systematic searches in MEDLINE (Ovid version), targeted internet searches (Google Scholar) and snowball searches. A two-stage screening process (titles/abstracts/keywords and full-texts) will use specified exclusion/inclusion criteria to identify eligible articles. Data on how modifications are i) defined, ii) perceived, and iii) classified, and iv) views on how modifications should be measured and reported, will be extracted verbatim. Inductive thematic analysis will be applied to extracted data where appropriate. Results will be presented as a narrative summary including descriptive characteristics of included articles. Findings will inform a preliminary conceptual framework to facilitate the systematic reporting and sharing of modifications to novel procedures and devices.

**Highlights:**

- This work will generate an in-depth understanding of how modifications are currently defined, perceived and classified, and views on how they may be reported, in the context of surgical innovation.
- Rigorous and comprehensive search methods will be applied to identify a wide range of diverse data sources for inclusion in the review.
- A summary of existing relevant literature on modifications is a necessary step to inform development of a framework for transparent, real-time reporting and sharing of modifications in future studies of innovative invasive procedures/devices.

## 1. Introduction

### 1.1 Background

Surgical innovation has undoubtedly led to dramatic improvements in patient care. Unstandardised introduction of surgical procedures and devices, however, has the potential to cause significant patient harm [1,2]. This is particularly true in the early stages of surgical innovation when new techniques or technologies are still being modified and refined before they are optimised [3]. Current ways of reporting and sharing important incremental learning arising from each case, however, are informal and insufficient [4]. Consequently, individual surgeons may simultaneously refine the same technical steps, or even repeat ineffective or harmful modifications [5]. This can lead to delays in uptake of promising innovation and may critically increase the risk of avoidable patient harm [6].

Systematic monitoring of modifications is crucial to evaluating new procedures/devices during their introduction into clinical practice and before definitive evaluation in randomised controlled trials [7]. The importance of modifications has been recognised in a recent core outcome set developed for early phase surgical studies (the COHESIVE Study), with a ‘modifications’ domain agreed as one of the core outcome domains to be measured and reported in all studies [8]. Measurement and reporting of modifications in current empirical studies of surgical innovation is, however, heterogenous and lacks detail [9,10,11]. Standardisation of reporting and sharing of modifications is therefore integral to the safe and efficient introduction of new procedures/devices into clinical practice.

There is now an urgent need to define measurement and reporting of modifications. No agreed definition of what constitutes a modification to an evolving surgical procedure currently exists. Likewise, guidance on how modifications should be measured or classified to aid systematic reporting and sharing of modifications is lacking [9,12]. A framework is needed to facilitate transparent reporting and sharing of modifications in future studies of innovative invasive procedures/devices. It can provide surgeon innovators, device manufacturers and trialists with a standardised tool to be used throughout the innovation lifecycle to promote their efficient evaluation. Consequently, a necessary step to informing such a reporting framework is to examine and summarise existing relevant literature on modifications.

### 1.2 Aim and objectives

This systematic scoping review aims to examine how modifications to innovative surgical procedures/devices might be defined, perceived and classified, including views on how they should be measured and reported. Specific objectives of this scoping review are to identify literature in the context of innovative invasive procedures and devices that provide any existing:

- scientific or descriptive definitions of modifications;
- perceptions of modifications;
- classification systems, taxonomies or typologies for categorising modifications; and
- views and opinions on methods for measuring and reporting modifications.

### 1.3 Definitions

An invasive procedure is defined as “one where purposeful/deliberate access to the body is gained via an incision, percutaneous puncture, where instrumentation is used in addition to the puncture needle, or instrumentation via a natural orifice. It begins when entry to the body is gained and ends when the instrument is removed, and/or the skin is closed. Invasive procedures are performed by trained healthcare professionals using instruments, which include, but are not limited to, endoscopes, catheters, scalpels, scissors, devices and tubes” [13].

There is no agreed definition of innovative invasive procedures [14,15,16], and no validated methods to identify phase of evaluation retrospectively in published literature. Innovative invasive procedures were therefore defined as those where authors self-report an invasive procedure as “new” or “modified”, corresponding to IDEAL phases 1 and 2a [17,18].

## 2. Materials and methods

**Figure 1:**
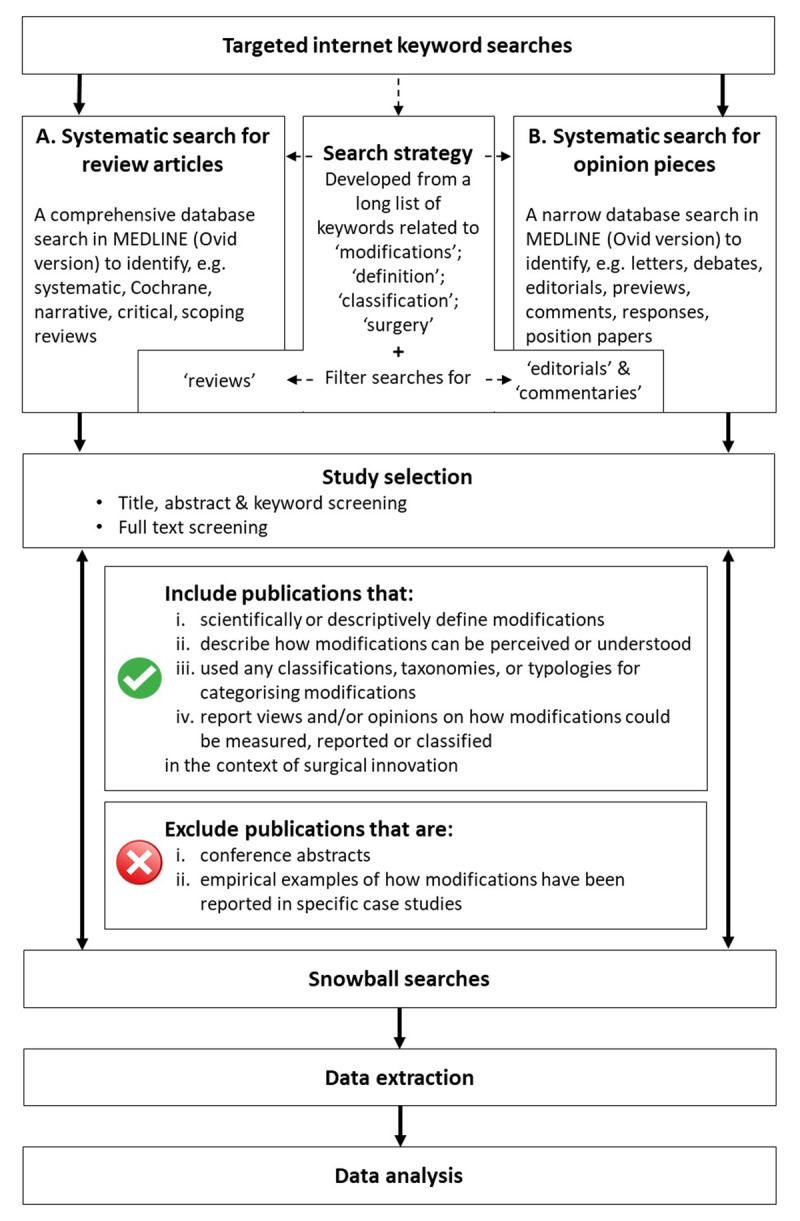
Study flow chart.

A scoping review was identified as a suitable method for mapping a complex topic where no prior comprehensive investigation has been performed [19,20,21]. This study will be conducted in accordance with the PRISMA guidelines for scoping reviews (PRISMA-ScR) and will adhere to established scoping review frameworks [20,21,22]. A study flow chart is presented in ***Figure 1***.

Ethical approval is not required for this scoping review.

### 2.1 Identifying relevant records

#### 2.1.1 Data sources

Two specific, distinct publication types of interest have been identified a priori by the research team. It is hypothesised that i) review articles and ii) opinion pieces will represent the most useful data sources for this review. This is because modifications are more likely to be discussed in greater detail outside of the existing traditional reporting parameters of surgical research studies and case reports. In addition, review articles and opinion pieces are thought to hold valuable information about desired reporting practices in view of deficient reporting practices in empirical studies [9,10,11].

A wide range of review articles will be considered and may include, but are not limited to, systematic literature reviews (including Cochrane reviews), narrative, critical and scoping reviews. Opinion pieces will be described as published reports or expressions of “original and personal views” and are typically, but not limited to, letters, debates, editorials, previews, comments, responses or position papers [23].

Tailored, specific searches are required to identify a manageable number of potentially relevant articles. Therefore, two systematic database searches will be undertaken to identify review articles and opinion pieces, respectively. These searches will be supplemented with snowball searches to ensure a comprehensive approach to identifying relevant literature. Details for each search are described below.

#### 2.1.2 Systematic database searches

Previous reviews undertaken by the study team have found that the use of search terms related to “modification” and “invasive procedures” retrieve a large number of irrelevant records. Development of a sufficiently focused search strategy is needed to avoid retrieving an unmanageable number of records for full text screening. Therefore, targeted internet keyword searches will be used to inform a comprehensive search strategy to apply to the MEDLINE library catalogue database. Targeted internet searches will be performed in Google Scholar using keywords related to “modifications”, “definition”, “classification” and “invasive procedures” to identify relevant literature that reports definitions, perceptions and/or classifications of modifications. Identified articles will be read in full to derive further relevant keywords to be added to a long list of search terms. The long list will inform development of a comprehensive database search strategy which will be refined through discussions with the with the study team (consisting of surgeons, methodologists, trialists) and in collaboration with an expert subject librarian. Two separate searches will then be performed in MEDLINE (Ovid version) to identify records relevant to the two distinct publication types of interest (described below). Unique aspects inherent to each publication type will be considered when applying the search strategy. Searches will be limited to publications in English language and to studies in humans only.

##### A. Systematic search for review articles

A comprehensive search will be developed to identify all potentially relevant review articles, which can subsequently be narrowed down by screening titles and abstracts. This search will include a modified, previously validated search filter for reviews in order to identify relevant publications classified as reviews [24]. Specific changes to the search strategy will include the addition of the word “review*” and “narrative” as well as the omission of keywords related to meta-analyses, as these are presumed unlikely to yield relevant results. To reduce the number of potential records, publication dates will be limited to articles published between 2010 and 2021 to ensure the search focuses on contemporary literature that is more likely to provide information about current descriptions of modifications.

##### B. Systematic search for opinion pieces

A targeted search for published opinion pieces is necessary due a lack of abstracts available and/or detail provided in any abstracts for these types of publications, meaning screening for eligibility will be resource intensive. As a result, full-text articles will likely need to be obtained for the majority of opinion pieces. A more detailed search will be developed (e.g. using narrower proximity operators and/or fewer keywords), to obtain fewer results and limit the number of records necessary to be screened. Search filters for editorials and commentaries will be applied. No limits to publication date will be applied because this search is expected to produce a smaller number of potential records.

#### 2.1.3 Snowball searches

Backwards and forwards searching of reference lists (snowball searches) will be applied to all eligible records until no new relevant publications will be identified. This will detect records which may have been missed due to lack of indexing terms [25].

### 2.2 Inclusion criteria

Included will be any relevant review article or opinion piece published in a peer-reviewed journal that discusses modifications in the context of innovative invasive procedures/devices. Specifically, publications will be included that:

- scientifically or descriptively define modifications (study objective i), or
- describe how modifications may be perceived or understood (study objective ii), or
- used any classifications, taxonomies, or typologies for categorising modifications (study objective iii), or
- report views and/or opinions on how modifications could be measured or reported (study objective iv)

Studies discussing modifications to accompanying (concomitant) interventions (e.g. drains, dressings or analgesia) or changes to patient selection will also be included as these have been identified as relevant aspects of modifications in previous studies [4,8].

### 2.3 Exclusion criteria

The following publications are outside the scope of this work and will be excluded:

- conference abstracts due to the lack of in-depth information available
- empirical examples of how modifications have been reported in specific case studies (e.g. technical steps of a modification during surgical procedure or use of a device)

Other exclusion criteria are:

- Non-English publications
- Publications outside the years 2010–2021 (systematic search A only)

### 2.4 Study selection

Studies identified through the search process will undergo a two-stage study selection process.

First, titles, abstracts and keywords will be screened independently by two reviewers. Keyword screening is included in the first stage because it is anticipated that abstracts will hold less detail about modifications and/or may not use a common structure (in particular opinion pieces). Full texts will be retrieved where eligibility is confirmed, uncertain or where no abstract is presented.

Decisions on inclusion/exclusion during the first stage of study selection are expected to require a degree of subjective interpretation because titles, abstracts and keywords are expected to hold limited information about modifications with which eligibility can be assessed. Therefore, initial additional steps are planned to ensure consistency of study selection approach between the two independent reviewers. A random selection of a small number of studies will be used to (i) independently screen titles, abstracts and keywords to decide whether records are included, excluded or uncertain; (ii) discuss and investigate reasons for discrepancies of decisions, (iii) jointly screen full texts of remaining uncertain records to assess final eligibility, which will lead to refinements of study selection approach. This process will be repeated until reviewer agreement of independent inclusion/exclusion decisions has reached sufficient levels (80%).

Second, full texts will be screened by the same reviewers to assess eligibility of identified records. Each reviewer will screen half (50%) of all retrieved publications to decide their inclusion or exclusion in this review. Articles deemed eligible for inclusion and those where inclusion is uncertain will be discussed between the two reviewers to reach a decision. In addition, a third independent reviewer, will subsequently assess eligibility of all articles discussed between the two reviewers.

Reasons for exclusion will be documented at each screening stage and will follow a predefined order of priority (1; not related to surgery, 2; not a review article or opinion piece, 3; not related to modifications, 4; no information on definition/perception/classification/reporting of modifications). Double-screening will be carried out for at least 20% of title and abstracts and 10% of full text articles. Any discrepancies will be discussed between the three reviewers and any further disagreements will be arbitrated in discussions with the wider study team. The process of study selection will be reported in line with PRISMA-ScR guidelines [26].

### 2.5 Data extraction

A study-specific data extraction form in line with the research objectives will be developed by the research team. An initial proforma will be piloted with a small number of articles and iteratively refined to ensure all emerging relevant detail is comprehensively captured. The final data extraction form will be applied to all included articles. Information related to publication characteristics, details about author(s) and affiliations and conflict of interest statements will be extracted. Detail as to whether the article discusses surgical procedures, devices or both will also be captured to enable potential sub-group analysis. Any details related to modifications and their definition, perception, classification or views on their measurement and reporting will be extracted verbatim. At least 10% of included articles will undergo initial double data extraction. These will be compared and discussed to ensure consistency of the approach to data extraction. Data extraction will be conducted independently by two reviewers.

### 2.6 Data analysis

Study characteristics will be summarised using descriptive statistics and presented in a table. Data relating to study characteristics will be grouped into categories where appropriate. Categorisation might be based on existing guidelines when available (e.g. national statistics country classifications will be used to categorise geographical origin of included articles) or will be finalised in discussions with the wider team wherever necessary.

Verbatim text will be analysed using inductive thematic analysis and will be guided by the study objectives [27]. The extent of sub-group analyses will be led by the data. Analyses may be conducted, for example, separately for information categories (e.g. definitions, perceptions, classifications of modifications, views on how to measure and report them) and for article context (e.g. procedures, devices). Two reviewers (SH, CH) will independently perform analyses and will discuss interim findings during regular meetings. Data familiarisation will be achieved through reading and re-reading extracted data. Initial codes will be assigned to the textual data and discussed between the reviewers. Thematic structures for analyses will be developed jointly between the two reviewers and iteratively refined as new themes emerge from the data. Consensus discussions within the wider team will agree on a final thematic structure. Themes will be presented in a narrative summary. Where thematic analysis is not appropriate (e.g. any identified classification systems, taxonomies or typologies), data will be descriptively summarised and presented in tabulated format.

No quantitative analyses are planned as is it not expected that opinion pieces and review articles will yield data appropriate for this type of synthesis.
